# A Major and Stable Quantitative Trait Locus *qSS2* for Seed Size and Shape Traits in a Soybean RIL Population

**DOI:** 10.3389/fgene.2021.646102

**Published:** 2021-04-16

**Authors:** Giriraj Kumawat, Donghe Xu

**Affiliations:** ^1^Biological Resources and Post-Harvest Division, Japan International Research Center for Agricultural Sciences, Tsukuba, Japan; ^2^Crop Improvement Section, ICAR-Indian Institute of Soybean Research, Indore, India

**Keywords:** soybean, seed size, seed shape, seed yield, quantitative trait loci, stable QTLs, *qSS2*

## Abstract

Seed size and shape traits are important determinants of seed yield and appearance quality in soybean [*Glycine max* (L.) Merr.]. Understanding the genetic architecture of these traits is important to enable their genetic improvement through efficient and targeted selection in soybean breeding, and for the identification of underlying causal genes. To map seed size and shape traits in soybean, a recombinant inbred line (RIL) population developed from K099 (small seed size) × Fendou 16 (large seed size), was phenotyped in three growing seasons. A genetic map of the RIL population was developed using 1,485 genotyping by random amplicon sequencing-direct (GRAS-Di) and 177 SSR markers. Quantitative trait locus (QTL) mapping was conducted by inclusive composite interval mapping. As a result, 53 significant QTLs for seed size traits and 27 significant QTLs for seed shape traits were identified. Six of these QTLs (*qSW8.1, qSW16.1, qSLW2.1, qSLT2.1, qSWT1.2*, and *qSWT4.3*) were identified with LOD scores of 3.80–14.0 and *R*^2^ of 2.36%–39.49% in at least two growing seasons. Among the above significant QTLs, 24 QTLs were grouped into 11 QTL clusters, such as, three major QTLs (*qSL2.3*, *qSLW2.1*, and *qSLT2.1*) were clustered into a major QTL on Chr.02, named as *qSS2*. The effect of *qSS2* was validated in a pair of near isogenic lines, and its candidate genes (*Glyma.02G269400, Glyma.02G272100, Glyma.02G274900, Glyma.02G277200*, and *Glyma.02G277600*) were mined. The results of this study will assist in the breeding programs aiming at improvement of seed size and shape traits in soybean.

## Introduction

Soybean [*Glycine max* (L.) Merr.] is one of the world’s most economically important food and oil crops. It is a rich source of edible oil and protein, and provides substrate for several food and industrially important products (Lam et al., 2010; Kumawat et al., 2016). Increasing yield and appearance quality of soybean seed are primary breeding objectives. Seed yield per unit area is the product of the number of seeds per unit area and seed weight. Seed size is highly correlated with seed weight, and thus a primary breeding target to improve seed yield (Xu et al., 2011; Li et al., 2019). Seed size traits such as length (SL), width (SW), thickness (ST), and single seed weight (SSW), and seed shape traits such as length-to-width (SLW), length-to-thickness (SLT) and width-to-thickness (SWT) ratios, are important determinants of soybean seed appearance quality and yield (Xu et al., 2011; Hina et al., 2020).

Seed size and shape traits in soybean are not only major components of seed yield, but also important morphological traits in determining commercial value of soybean seed in international trade (Wilson, 1995; Liang et al., 2005; Cui and Xuan, 2007). Different types of soy-based food products also require a specific type of soybean seed shape and size (Cui et al., 2004). For example, large-seeded soybean varieties are used for the preparation of green soybean (edamame), boiled soybean (nimame), soymilk, soy nuts, and soybean curd (tofu), while small seeded ones are used for natto and soy sprouts (Liang et al., 2016; Teng et al., 2017; Wu et al., 2018).

Soybean seed size and shape traits are quantitatively inherited and influenced by genetic and environmental factors (Hu et al., 2013; Wang et al., 2015; Cui et al., 2020), making it difficult and time-consuming to improve these traits using traditional breeding methods (Hao et al., 2012). The genetic control of quantitatively inherited traits, such as seed size and shape traits, can be dissected and genomic positions of the controlling loci can be identified using molecular markers (Tanksley, 1993; Sun et al., 2012). These quantitative trait loci (QTLs) can be used in soybean breeding for introgression and selection of favorable alleles of various seed size and shape traits to facilitate the development of new soybean varieties with seed size and appearance tailored for each distinct endeavor (Allen, 1994; Cao et al., 2017; Cui et al., 2020).

In soybean, QTLs for seed size and shape traits have been identified using simple sequence repeats (SSRs) and single nucleotide polymorphisms (SNPs) markers (Hoeck et al., 2003; Salas et al., 2006; Xu et al., 2011; Hu et al., 2013; Niu et al., 2013; Kato et al., 2014; Liang et al., 2016; Fang et al., 2017; Yang et al., 2017; Fujii et al., 2018; Li et al., 2019, 2020; Cui et al., 2020; Hina et al., 2020). Many QTLs have been identified for seed size and shape traits in soybean, but our understanding of their genetic architecture for practical application in soybean breeding is still limited because most of the QTLs are population and environment specific (Hina et al., 2020). Although association mapping uses diverse natural populations, yet the identified loci must be characterized and validated for their allelic effect and environmental stability in the potential genetic sources used in the breeding program. This study further explored the genetic architecture of seed size and shape traits in soybean using a recombinant inbred line (RIL) population and identified six stable QTLs for seed size and shape traits with large effect phenotypic contribution across multiple years. A major genomic locus harboring three major QTLs was validated in residual heterozygous line (RHL)-derived near isogenic lines (NILs).

## Materials and Methods

### Plant Material and Phenotypic Evaluation

A RIL population of 94 RILs was used to map QTLs for seed size and shape. The RIL population was derived from a cross between “K099” cultivar having small and round seed shape and “Fendou 16” cultivar having large and oblong seed shape. K099 is a Korean cultivar and Fendou 16 (PI574476A) is a cultivar from Shanxi, China. K099 was provided by the National BioResource Project (*Lotus japonicus* and *G. max*)^1^, and Fendou 16 was provided by the US National Plant Germplasm System (NPGS)^2^. The RIL population (F_6_) was developed from the F_2_ generation by the single-seed descent method without selection during the generation advancement.

The RIL population was grown in 2012, 2016, and 2017 at the farm of Japan International Research Center for Agricultural Sciences, Tsukuba, Ibaraki, Japan. Each RIL was grown in a 3-m single row plot spaced 60 cm between rows and 20 cm between plants. Seeds were harvested as plot bulk and a total of 20 seeds randomly selected from each RIL, were used for the trait measurement from three growing environments. The RIL population was phenotyped for four seed size traits namely SSW (g), SL, SW, and ST (mm) using a digital Vernier caliper, as described in Niu et al. (2013). Three seed shape traits namely SLW, SLT, and SWT, were calculated from the seed size traits for each RIL (Niu et al., 2013).

### DNA Marker Analysis and Linkage Map Construction

Amplicon sequence-based genotyping was performed using genotyping by random amplicon sequencing-direct (GRAS-Di) technology at Eurofins Genomics, Tokyo (Enoki and Takeuchi, 2018). Libraries were constructed for GRAS-Di, according to the protocol described in Hosoya et al. (2019). Sequencing of the libraries was conducted using the Illumina HiSeq4000. Marker identification was performed using GRAS-Di software version 1.0.5 (Toyota, Aichi, Japan). The software evaluates marker quality according to the empirical criteria of genotyping reproducibility identified based on the number of reads and reproducibility of genotyping (presence and absence of reads) over samples (Patent ID P2018-42548A). Genotype of a RIL sample was determined based on the number of reads, and presence or absence of respective read compared to the reference sample (Fendou 16). The identified GRAS-Di markers were tested for 1:1 segregation ratio using the *Chi-*square test. Markers (reads) identified in this way were mapped to soybean genome Wm82.a2.v1, using BWA-MEM version 0.7.12 (Li, 2013).

Other than GRAS-Di markers, classical SSR markers were also genotyped in the RIL population. Publically available soybean SSRs on SoyBase^3^ were amplified (∼600) for polymorphism analysis between the parents of RILs as per methods given in Liu et al. (2018). PCR amplification was performed in a final volume of 20 μl with 10 ng of template DNA, 10 pmol forward and reverse primers, and 10 μl Quick Taq^TM^ HS DyeMix (Toyobo, Tokyo, Japan). The PCR reaction was performed over 35 cycles of 30 s at 94°C, 30 s at 56°C, and 30 s at 72°C followed by an extension of 5 min at 72°C. Amplified products were separated on 8.0% polyacrylamide gel and stained with ethidium bromide. The banding pattern was documented using a Pharos FX Molecular Imager (Bio-Rad, Tokyo, Japan). A total of 229 polymorphic SSR markers were genotyped in the RILs.

Redundant markers with high collinearity were filtered out using the bin functionality of QTL IciMapping software (Meng et al., 2015). Non-redundant SSR and GRAS-Di markers were used for linkage map construction using MapDisto version 2.0 software (Heffelfinger et al., 2017). A logarithm of odds (LOD) score of 6.0 and maximum recombination fraction of 0.2 estimated using the classical method was used to group of markers. Kosambi mapping function and the seriation II method were used for ordering markers.

### Data Analysis and QTL Mapping

Phenotypic data were analyzed in Excel 2016 software (Microsoft, Redmond, WA, United States). Correlations among traits were assessed using Pearson’s correlation coefficient and visualized using the “corrplot” package in R (Wei and Simko, 2017). Trait values between genotype pairs were compared for significance by Student’s *t*-test. The inclusive composite interval mapping (ICIM) method was used to identify seed size and shape QTLs, using the QTL IciMapping software (Meng et al., 2015). The significance for the declaration of QTL was estimated from 1,000 permutation tests. A LOD score corresponding to an experiment-wise threshold of *P* = 0.05 was used to declare a QTL as significant. Composite interval mapping in Windows QTL Cartographer v2.5 (Wang et al., 2012) was used to confirm the QTL results. Confidence interval of QTLs were determined using a 1-LOD support interval as a 95% confidence interval (Lander and Botstein, 1989). Neighboring QTLs of different seed size and shape related traits with overlapping confidence intervals were grouped into QTL clusters. The map positions of QTLs in the QTL clusters were depicted on the linkage map using MapChart software (Voorrips, 2002).

### Development of NILs and *qSS2* Validation

Two NILs, NILs-F, and NILs-K, were selected from the progenies of a self-pollinated F_6_ RIL, RIL98, which was heterozygous at QTL cluster *qSS2*. Homozygous plants with the Fendou 16 and K099 genotypes at *qSS2* were selected from the RIL98 progenies based on SSR markers BARCSOYSSR_02_1667 and Sat_415. The genomic background difference between NIL pair was analyzed using 122 SSR markers selected from 20 soybean chromosomes. These contrasting NILs, NILs-F, and NILs-K, were used to confirm the effect of *qSS2*. The NILs were grown in 2018 and 2019 under similar agronomic conditions as the RIL population. Each NIL was phenotyped for seven seed size and shape traits (SL, SW, ST, SSW, SLW, SLT, and SWT) in three replicates of 20 seeds each.

### Candidate Gene Mining in the QTL Cluster *qSS2*

Predicted genes in the physical genomic interval of *qSS2* and their annotations information were downloaded from SoyBase (see text footnote 3). A freely available RNA-Seq data set on SoyBase, which includes seven seed development stages (seed_10DAF, seed_14DAF, seed_21DAF, seed_25DAF, seed_28DAF, seed_35DAF, and seed_42DAF, where DAF stands for days after flowering), was used to download expression data for the predicted candidate genes to analyze the expression of these genes among different tissue samples and developmental stages (Severin et al., 2010). A heatmap was constructed by using TBtools software to visualize the relative expression patterns of the predicted candidate genes in different tissue samples (Chen et al., 2020). Candidate genes were mined based on the gene annotations, expression data, and available literature.

## Results

### Phenotypic Variation for Seed Size and Shape Traits

**FIGURE 1:**
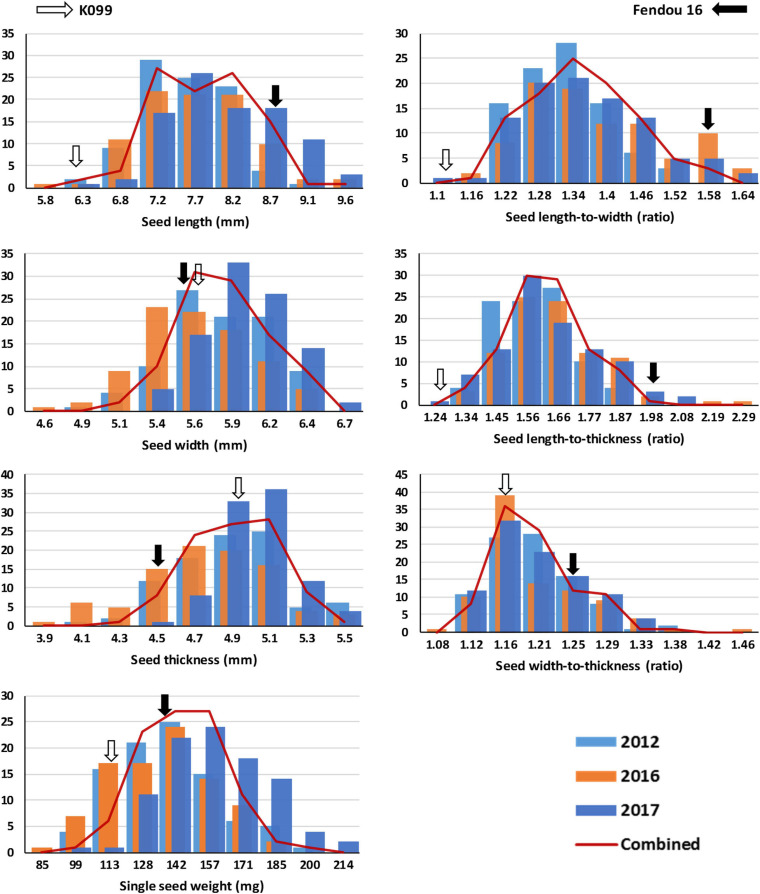
Frequency distribution of seed length (SL), seed width (SW), seed thickness (ST), single seed weight (SSW), and ratios of seed length-to-width (SLW), seed length-to-thickness (SLT), and seed width-to-thickness (SWT) in K099 × Fendou 16 recombinant inbred line (RIL) population (*n* = 94) grown across multiple years (2012, 2016, and 2017). Trend lines show the average of 3-year environments data. Arrows represent the mean value of the corresponding parent genotype. The horizontal and vertical axes represent the trait value and number of RILs, respectively.

**FIGURE 2:**
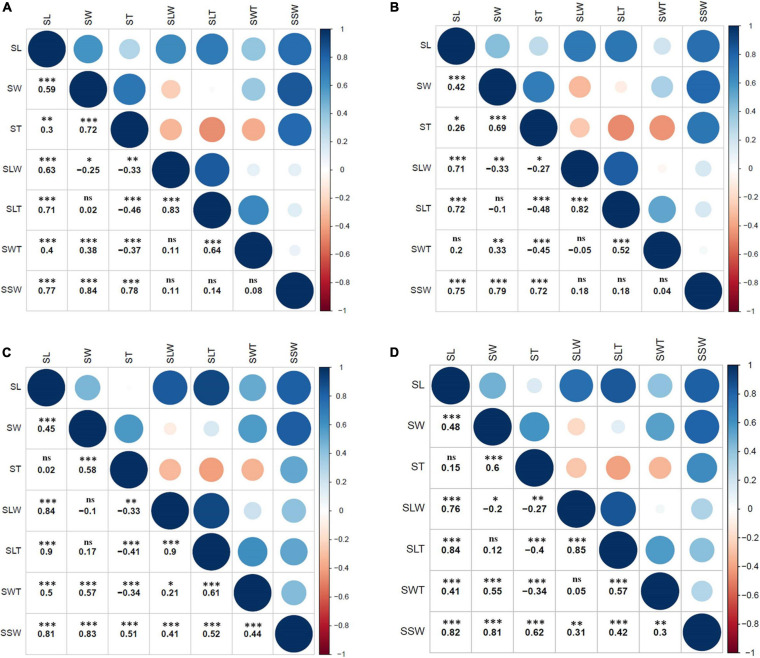
Pearson’s correlation analysis of seven seed size and shape traits among RILs (*n* = 94) within or across environments; **(A)** 2012, **(B)** 2016, **(C)** 2017, and **(D)** combined (mean values of 3 years). ^∗^, ^∗∗^, and ^∗∗∗^ indicate significant correlations at the 5%, 1%, and 0.1% level, respectively; ns indicates no significant correlation at the 5% level.

The two parental genotypes of the RIL population, K099 and Fendou 16, significantly differ for all seed size and seed shape traits (*P* < 0.01, Supplementary Table 1). Fendou 16 seeds are larger with trait values 21.5% (SSW), 29.3% (SL), 30.7% (SLW), 36.7% (SLT), and 8.7% (SWT) higher compared to K099, which has an ST value 11.3% higher than Fendou 16. The absolute values of kurtosis and skewness were <1 for most traits in all years, except for SLT and SWT in 2016 and ST in 2017, indicating typical quantitative inheritance of seed size and shape traits in RILs (Supplementary Table 2). Higher and lower values than means of the parental genotypes was observed for most of the evaluated traits indicating transgressive segregation (Figure 1). Correlation analysis identified significant positive correlations between SL and all other seed size and shape traits, except ST and SWT in 2017 and 2016, respectively (Figure 2). SSW was positively correlated with all seed size and shape traits in 2017 and combined environments data, but was not significant for three seed shape traits in 2012 and 2016. Significant negative correlations were observed between SW and SLW, and for ST with respect to SLW, SLT and SWT (Figure 2).

### Marker Identification and Linkage Map Construction in 94 RILs

Genotyping by random amplicon sequencing, direct of 94 RILs and two parents yielded an average of 6,050,345 paired-end reads for each line generating 611 Mb sequence data per line. After filtering markers with low quality and segregation distortion (1:1, *P* > 0.01), 4,366 high quality polymorphic markers (reads) were obtained. These 4,366 GRAS-Di markers and 229 SSR markers were used for binning to remove redundant markers with high collinearity, resulting in 2,010 unique markers. These 2,010 unique markers were used for linkage map construction. Of these, 1,662 markers (1,485 GRAS-Di and 177 SSRs) were correctly assigned to 20 soybean chromosomes (Supplementary Table 3). Chromosome 12 had the fewest markers (*n* = 50), and Chromosome 9 had the most (*n* = 119). Most markers showed collinearity with the physical map, with exception of a few segments on Chr.01, Chr.09, Chr.18, and Chr.20 (Supplementary Table 3). The total map length spans 4,464 cM with an average of 2.68 cM distance per marker (Supplementary Table 4). The longest linkage group was for Chr.13 with a 304.2 cM genetic map distance, and the shortest was for Chr.14 with a 154.2 cM map distance.

### QTLs for Seed Size Traits

Inclusive composite interval mapping identified 53 QTLs, distributed on 17 chromosomes, for four seed size traits in RILs (Table 1). Of these, 19 QTLs were identified for SL, 15 for SW, 7 for ST, and 12 for SSW. The 19 QTLs identified for SL explained 4.39–20.86% of phenotypic variance (PV), 10 of which were major QTLs accounting for >10% phenotypic variation (Table 1). Two major QTLs for SL, *qSL2.2* and *qSL2.3*, were identified in the nearby genomic regions for the growing environments of 2017 and 2016, respectively, while *qSL2.3* was also detected in the combined environments data. The *qSL2.3* was detected with a LOD score of 14.1 between BARCSOYSSR_02_1686 and BARCSOYSSR_02_1697 for the combined environments data and explained 19.85% of phenotypic variance (PV) for SL in the RIL population. The additive allele of Fendou 16 at *qSL2.3* increased SL by 0.26 mm in the combined environments data. One QTL, *qSL10.1*, was identified in 2016, as well as for the combined environments data, explaining 20.86% and 14.06% of PV, respectively. Two QTLs, *qSL11.1*, and *qSL11.2*, identified for combined environments data and 2012, respectively, are present in close proximity and seem to be the same QTL. Similarly, *qSL13.2* and *qSL13.3* were also detected in close proximity for combined environments data and 2017, respectively.

**TABLE 1:** QTLs identified for seed-size traits in K099 × Fendou 16 RIL population across multiple years.

**Trait^#^**	**QTL^a^**	**Position^*b*^**	**Flanking**	**LOD^d^**	**PVE^e^**	**Add.^f^**	**Confidence**	**Env.^h^**	**References^i^**
		**(cM)**	**markers^c^**		**(%)**		**interval^g^ (cM)**		
SL	*qSL1.1*	181	Chr01_51855286 – Sat_414	3.6	11.70	–0.21	176.5–184.5	2016	
	*qSL2.1*	25	Chr02_4637515 – Chr02_4901668	6.1	15.07	–0.22	24.5–27.5	2012	Niu et al. (2013)
	*qSL2.2*	196	Sat_183 – Chr02_45158577	8.0	15.30	–0.31	193.5–198.5	2017	
	*qSL2.3*	204	BARCSOYSSR_02_1697 – Chr02_46197997	4.9	16.65	–0.25	202.5–205.5	2016	
		202	BARCSOYSSR_02_1686 – BARCSOYSSR_02_1697	14.1	19.85	–0.26	201.5–202.5	Combined	
	*qSL4.1*	157	Chr04_47507331 – Chr04_47464069	3.9	8.72	–0.16	156.5–158.5	2012	
	*qSL7.1*	42	Chr07_5763313 – Chr07_5403784	4.7	8.38	0.23	40.5–42.5	2017	
	*qSL9.1*	235	BARCSOYSSR_09_1654 – Chr09_49212976	5.5	12.76	–0.20	231.5–236.5	2012	
	*qSL10.1*	252	Chr10_45097961 – Chr10_46431380	5.8	20.86	–0.28	244.5–254.5	2016	Xu et al. (2011)
		251	Chr10_45097961 – Chr10_46431380	10.2	14.06	–0.22	246.5–254.5	Combined	
	*qSL11.1*	43	Chr11_4842436 – Chr11_4833882	5.0	5.67	0.14	42.5–46.5	Combined	
	*qSL11.2*	49	Chr11_6273527 – Sat_261	4.6	10.39	0.18	47.5–50.5	2012	
	*qSL13.1*	115	Chr13_17185244 – Chr13_17189402	5.4	6.14	–0.14	114.5–115.5	Combined	Hina et al. (2020)
	*qSL13.2*	185	Chr13_29854639 – Satt114	11.5	15.39	0.23	183.5–186.5	Combined	
	*qSL13.3*	191	Chr13_30864439 – Chr13_31700804	5.1	8.88	0.24	190.5–192.5	2017	
	*qSL15.1*	230	Satt369 – Chr15_48645827	6.5	12.0	–0.27	228.5–232.5	2017	
	*qSL16.1*	214	Chr16_36834289 – Chr16_36721834	3.9	4.85	–0.13	210.5–215.5	Combined	
	*qSL17.1*	30	Chr17_4841475 – Chr17_5807297	3.5	7.91	–0.16	27.5–35.5	2012	
	*qSL17.2*	127	Chr17_27179004 – Chr17_18506669	6.1	11.20	–0.27	126.5–128.5	2017	
	*qSL19.1*	80	Chr19_8931895 – Chr19_11670696	4.0	4.39	–0.12	79.5–80.5	Combined	
	*qSL20.1*	116	Chr20_31337280 – Chr20_34302170	6.1	7.68	–0.16	112.5–117.5	Combined	
SW	*qSW1.1*	194	Chr01_52403587 – Chr01_53725721	4.9	9.44	–0.13	190.5–194	2016	Cui et al. (2020)
	*qSW2.1*	221	Satt271 – Chr02_47977562	4.3	1.23	0.07	220.5–221	2017	
	*qSW4.1*	52	Chr04_6584200 – Chr04_6873174	3.5	8.34	–0.09	50.5–54.5	2012	
	*qSW5.1*	106	Chr05_29039821 – Chr05_31884183	4.0	1.11	0.06	103.5–106.5	2017	
	*qSW5.2*	154	Chr05_35848263 – Chr05_36437168	5.1	9.48	0.13	152.5–156.5	2016	
	*qSW5.3*	160	Chr05_37341943 – Chr05_38019119	6.5	16.9	0.13	158.5–161.5	2012	
	*qSW5.4*	195	Chr05_39358284 – Satt225	5.9	8.32	0.09	192.5–196.5	Combined	
	***qSW8.1***	39	BARCSOYSSR_08_0442 – Chr08_8508402	4.4	8.35	–0.12	36.5–42.5	2016	Niu et al. (2013); Liang et al. (2016), Hina et al. (2020)
		34	Chr08_5524528 – BARCSOYSSR_08_0382	7.8	2.36	–0.09	32.5–36.5	2017	
		38	BARCSOYSSR_08_0382 – BARCSOYSSR_08_0442	8.9	12.92	–0.11	34.5–40.5	Combined	
	*qSW11.1*	50	Sat_261 – Chr11_5735979	4.0	9.34	0.10	49.5–51.5	2012	
		51	Chr11_5735979 – Chr11_6215680	4.5	5.79	0.08	50.5–51.5	Combined	
	*qSW11.2*	57	Chr11_6215680 – Chr11_6798953	6.7	2.21	0.09	53.5–59.5	2017	
	*qSW12.1*	4	Chr12_159254 – Sat_200	5.2	6.97	0.08	2.5–4.5	Combined	
	*qSW13.1*	178	Chr13_26888971 – Chr13_27754596	37.8	26.87	–0.33	176.5–178.5	2017	
	*qSW13.2*	180	Chr13_28816766 – Chr13_29071614	42.6	35.21	0.38	179.5–180.5	2017	
	***qSW16.1***	210	Chr16_36296773 – Chr16_36530800	5.8	14.74	–0.12	208.5–210.5	2012	
		208	Satt431 – Chr16_36260749	10.4	21.47	–0.20	206.5–208.5	2016	
		208	Satt431 – Chr16_36260749	14.0	23.00	–0.16	206.5–208.5	Combined	
	*qSW19.1*	30	Chr19_3500167 – Sat_071	9.0	2.77	–0.10	29.5–31.5	2017	Xu et al. (2011); Niu et al. (2013)
		30	Chr19_3500167 – Sat_071	5.0	6.58	–0.08	29.5–31.5	Combined	
ST	*qST2.1*	125	Chr02_39060823 – Chr02_39119748	6.7	22.77	0.11	124.5–127.5	2017	
	*qST6.1*	182	Chr06_48563257 – Satt371	4.1	13.07	–0.08	179.5–186.5	2017	Salas et al. (2006); Hina et al. (2020)
	*qST9.1*	51	Chr09_11721965 – Chr09_20289378	3.6	15.79	0.10	49.5–51.5	2012	
	*qST12.1*	110	Chr12_34396438 – Chr12_34687484	3.9	13.42	–0.08	108.5–113.5	Combined	Hina et al. (2020)
	*qST15.1*	79	Chr15_9068274 – Chr15_9299151	3.7	11.08	0.07	77.5–80.5	Combined	
	*qST16.1*	113	Chr16_11518752 – Chr16_21245278	4.2	13.38	–0.08	111.5–115.5	Combined	
	*qST16.2*	124	Chr16_26789246 – Chr16_26960235	3.8	16.97	–0.10	122.5–125.5	2012	
SSW	*qSSW2.1*	217	Chr02_47839472 – Chr02_47824243	4.4	5.26	–5.99	216.5–218.5	2017	
	*qSSW5.1*	153	Chr05_35848263 – Chr05_36437168	6.9	17.63	10.54	151.5–153.5	2016	
	*qSSW9.1*	12	Chr09_3017176 – Chr09_3417491	3.9	9.72	–7.80	8.5–14.5	2016	
	*qSSW10.1*	168	Chr10_38608447 – Chr10_38881892	5.5	6.76	6.77	162.5–170.5	2017	
	*qSSW10.2*	243	Chr10_45097961 – Chr10_46431380	12.3	17.79	–11.07	241.5–246.5	2017	
		253	Chr10_45097961 – Chr10_46431380	4.1	10.98	–5.69	245.5–254.5	Combined	
	*qSSW12.1*	113	Chr12_34396438 – Chr12_34687484	3.9	11.13	–6.95	108.5–115.5	2012	
		114	Chr12_34687484 – Chr12_34872685	7.0	20.38	–7.80	111.5–115.5	Combined	
	*qSSW13.1*	76	Chr13_15111808 – Chr13_15130583	4.0	11.14	6.84	75.5–76.5	2012	
	*qSSW13.2*	180	Chr13_28816766 – Chr13_29071614	11.5	16.20	10.68	178.5–181.5	2017	
		180	Chr13_28816766 – Chr13_29071614	4.8	12.86	6.25	178.5–181.5	Combined	
	*qSSW15.1*	131	Chr15_15454204 – Chr15_15454297	4.5	10.13	8.02	129.5–133.5	2016	
	*qSSW17.1*	248	Chr17_39354538 – Sat_086	7.3	9.53	–8.09	245.5–250.5	2017	
	*qSSW20.1*	43	Chr20_19815936 – Chr20_30738097	4.5	13.61	–7.84	38.5–45.5	2012	
	*qSSW20.2*	117	Chr20_34302170 – Satt354	7.6	9.65	–8.20	115.5–118.5	2017	
		115	Chr20_31337280 – Chr20_34302170	3.8	11.04	–5.77	111.5–118.5	Combined	

*#SL, seed length; SW, seed width; ST, seed thickness; SSW, single seed weight. ^a^The QTL name is defined by the trait name, chromosome number and its order on the chromosome, stable QTLs detected in two or more environment are highlighted in bold. ^b^Position of peak likelihood of the QTL in cM. ^c^Right and left flanking markers of the significant QTL interval. ^d^The log of odds (LOD) value at the peak likelihood of the QTL. ^e^Phenotypic variance (%) explained by the QTL. ^f^Indicates additive effect. ^g^Confidence interval is the QTL support interval identified by 1-LOD drop from the peak LOD of QTL, in cM. ^h^Environment. ^i^References for the previously reported QTL(s) for corresponding seed size and shape traits in the physical genomic region of flanking markers.*

Fifteen QTLs were identified for the SW trait explaining 1.11% to 35.21% of PV across three environments (Table 1). Among them, two QTLs, *qSW8.1*, and *qSW16.1*, were stably expressed. Stable QTL, *qSW8.1* was identified in 2016 and 2017, as well as for combined environments data. Positive allele for SW at this locus was contributed by Fendou 16. Stable QTL *qSW16.1* was detected between 206.5 cM to 2010.5 cM, for 2012, 2016 and the combined environments data. PV explained by this stable QTL ranged from 14.74% to 23%, and positive allele for SW at this QTL was also contributed by Fendou 16. Interestingly two SW QTLs, *qSW13.1*, and *qSW13.2*, were detected in nearby genomic regions with large PV explained (26.87 and 35.21%, respectively) in 2017, but the QTL effect was found to be in the opposite direction (Table 1). The positive allele at *qSW13.1* was contributed by Fendou 16, whereas *qSW13.2*, is from K099.

Of the seven QTLs identified for ST, three (*qST12.1*, *qST15.1*, and *qST16.1*) were identified for combined environments data (Table 1). The major QTL *qST2.1* was detected in 2017, explaining 22.77% of PV. K099 contributed favorable allele at this major. QTLs *qST16.1* and *qST16.2* were in close proximity explaining 13.38% and 16.97% of PV for combined environments data and 2012, respectively. The positive alleles at both of these ST QTLs were contributed by Fendou 16.

Twelve QTLs were identified for SSW, explaining 5.26% to 20.38% of PV. Among them, *qSSW10.2, qSSW12.1, qSSW13.2*, and *qSSW20.2* were identified in one of the environments and combined environments data. Highest PV of 20.38%, was explained by *qSSW12.1* for combined environments data. Fendou 16 contributed positive alleles at seven SSW QTLs and K099 contributed positive alleles at five QTLs (Table 1).

Composite interval mapping of seed size traits identified 54 QTLs, 36 of which were shared with the QTLs identified by ICIM (Supplementary Tables 5, 6). Stable QTLs *qSW8.1* and *qSW16.1* were also detected at the same genomic position in CIM.

### QTLs for Seed Shape Traits

A total of 27 QTLs were identified for three seed shape traits which were distributed on 12 chromosomes (Table 2). Of these, four QTLs were identified for SLW, 13 for SLT and 10 for SWT. The four QTLs identified for SLW explained 6.91 to 39.49% of PV across various environments. One major and stable QTL *qSLW2.1*, was identified consistently at the same genomic region between map position 199.5 cM to 205.5 cM on Chr.02, in all of the 3 years growing environments as well as for combined environments data. *qSLW2.1* explained 29.04 to 37% of PV in the RILs across 3 years’ growing environments, and 39.49% of PV for the combined environments data. Positive allele at *qSLW2.1* was contributed by Fendou 16. Among three environment-specific QTLs, *qSLW8.1* and *qSLW9.1* were detected in the growing environment of 2012, and *qSLW10.1* was identified in 2016.

**TABLE 2:** QTLs identified for seed-shape traits in K099 × Fendou 16 RIL population across multiple years.

**Trait^#^**	**QTL^a^**	**Position^*b*^**	**Flanking**	**LOD^d^**	**PVE^e^**	**Add.^f^**	**Confidence**	**Env.^h^**	**References^i^**
		**(cM)**	**markers^c^**		**(%)**		**interval^g^ (cM)**		
SLW	***qSLW2.1***	204	BARCSOYSSR_02_1697 – Chr02_46197997	14.5	37.00	–0.051	203.5–205.5	2012	Niu et al. (2013)
		204	BARCSOYSSR_02_1697 – Chr02_46197997	12.7	39.48	–0.073	203.5–205.5	2016	
		201	BARCSOYSSR_02_1667 – BARCSOYSSR_02_1686	9.1	29.04	–0.068	199.5–202.5	2017	
		204	BARCSOYSSR_02_1697 – Chr02_46197997	15.4	39.49	–0.069	203.5–205.5	Combined	
	*qSLW8.1*	0	Chr08_2275213 – Chr08_2585878	5.5	10.79	–0.028	0–1.5	2012	
	*qSLW9.1*	15	Chr09_3417491 – Chr09_4747318	3.6	6.91	–0.022	12.5–19.5	2012	
	*qSLW10.1*	237	Satt581 – Chr10_45097961	4.9	12.14	–0.041	235.5–240.5	2016	Xu et al. (2011); Hina et al. (2020)
SLT	*qSLT1.1*	164	BARCSOYSSR_01_1420 – Chr01_50857719	5.1	13.45	–0.065	159.5–166.5	2016	
	***qSLT2.1***	202	BARCSOYSSR_02_1686 – BARCSOYSSR_02_1697	7.7	13.70	–0.046	200.5–202.5	2012	Fang et al. (2017)
		204	BARCSOYSSR_02_1697 – Chr02_46197997	7.7	20.68	–0.080	202.5–205.5	2016	
		201	BARCSOYSSR_02_1667 – BARCSOYSSR_02_1686	5.9	21.41	–0.079	198.5–202.5	2017	
		202	BARCSOYSSR_02_1686 – BARCSOYSSR_02_1697	10.6	23.21	–0.064	199.5–202.5	Combined	
	*qSLT8.1*	2	Chr08_2608524 – Chr08_2695499	4.0	6.41	–0.031	1.5–6.5	2012	Hina et al. (2020)
	*qSLT8.2*	111	Chr08_18327010 – Chr08_19032465	3.7	5.84	0.030	109.5–112.5	2012	
	*qSLT9.1*	53	Chr09_21284860 – Chr09_19460557	7.4	13.12	–0.044	52.5–54.5	2012	
	*qSLT9.2*	77	Chr09_11702533 – Satt178	6.0	11.53	–0.045	69.5–78.5	Combined	Niu et al. (2013)
	*qSLT10.1*	251	Chr10_45097961 – Chr10_46431380	5.7	11.93	–0.046	245.5–254.5	Combined	Xu et al. (2011); Hina et al. (2020)
	*qSLT10.2*	276	Chr10_48758210 – Chr10_49620085	3.6	8.55	–0.051	273.5–277.5	2016	
	*qSLT13.1*	117	Chr13_17205922 – Chr13_17558529	3.6	6.77	–0.034	116.5–117.5	Combined	Xu et al. (2011)
	*qSLT13.2*	185	Chr13_29854639 – Satt114	4.8	8.94	0.040	182.5–186.5	Combined	
	*qSLT15.1*	230	Satt369 – Chr15_48645827	4.1	14.03	–0.063	227.5–232.5	2017	
	*qSLT17.1*	16	Chr17_3515737 – BARCSOYSSR_17_0252	6.2	11.65	–0.042	13.5–18.5	2012	Hina et al. (2020)
	*qSLT18.1*	10	Chr18_2032040 – Chr18_2167574	7.5	13.28	–0.045	8.5–11.5	2012	
		10	Chr18_2032040 – Chr18_2167574	4.1	7.54	–0.036	9.5–11.5	Combined	
SWT	*qSWT1.1*	162	Chr01_50700241 – BARCSOYSSR_01_1420	7.7	20.06	–0.029	159.5–166.5	2016	
	***qSWT1.2***	182	Chr01_51855286 – Sat_414	4.3	11.25	–0.016	175.5–185.5	2012	
		183	Chr01_51855286 – Sat_414	4.1	15.77	–0.018	180.5–185.5	2017	
		181	Chr01_51855286 – Sat_414	11.8	21.84	–0.025	176.5–183.5	Combined	
	*qSWT4.1*	48	Chr04_5085005 – Chr04_6400800	7.9	14.58	–0.021	44.5–50.5	Combined	
	*qSWT4.2*	69	Chr04_7978929 – Chr04_8076913	4.3	15.64	–0.018	66.5–70.5	2017	
	***qSWT4.3***	144	Chr04_46382211 – Chr04_46508852	6.2	15.70	–0.019	143.5–144.5	2012	
		144	Chr04_46382211 – Chr04_46508852	3.8	8.73	–0.019	142.5–144.5	2016	
		144	Chr04_46382211 – Chr04_46508852	3.8	5.71	–0.013	142.5–144.5	Combined	
	*qSWT9.1*	193	Chr09_43070174 – Chr09_43203698	4.6	7.09	0.015	191.5–195.5	Combined	
	*qSWT13.1*	70	Chr13_14092162 – Chr13_14335705	6.0	14.77	–0.19	68.5−78.5	2012	
	*qSWT18.1*	35	Chr18_4239294 – Chr18_5003855	7.1	11.71	–0.019	33.5–36.5	Combined	
	*qSWT18.2*	74	Chr18_8011839 – Chr18_10444685	6.1	15.08	–0.025	71.5–74.5	2016	
	*qSWT19.1*	39	Chr19_4198272 – Chr19_3956525	4.7	17.25	–0.019	35.5–39.5	2016	
	*qSWT20.1*	224	Chr20_47084769 – Chr20_47875659	4.1	10.15	0.016	221.5–225.5	2012	

*^#^SLW, seed length to width ratio; SLT, seed length to thickness ratio; SWT, seed width to thickness ratio. ^a^The QTL name is defined by the trait name, chromosome number and its order on the chromosome, stable QTLs detected in two or more environment are highlighted in bold. ^b^Position of peak likelihood of the QTL in cM. ^c^Right and left flanking markers of the significant QTL interval. ^d^The log of odds (LOD) value at the peak likelihood of the QTL ^e^Phenotypic variance (%) explained by the QTL. ^f^Indicates additive effect. ^g^Confidence interval is the QTL support interval identified by 1-LOD drop from the peak LOD of QTL, in cM. ^h^Environment. ^i^References for the previously reported QTL(s) for corresponding seed size and shape traits in the physical genomic region of flanking markers.*

Thirteen QTLs were detected for SLT explaining 5.84 to 23.21% of PV (Table 2). A major and stable QTL *qSLT2.1*, was identified at a similar genomic region between map position 199.5 cM and 205.5 cM on Chr.02, in all 3 years’ growing environments and combined environments data. *qSLT2.1* explained 13.70 to 21.41% of the PV across the 3 years’ growing environments, and 23.21% of PV for the combined environments data. Positive allele at *qSLT2.1* was contributed by Fendou 16. *qSLT2.1* was identified in the same genomic region as *qSL2.3* and *qSLW2.1*, indicating pleiotropic control of these traits by this locus. *qSLT18.1* was detected in 2012 and for the combined environments data, explaining 13.28 and 7.54% of PV, respectively. Fendou 16 contributed favorable alleles at all SLT QTLs, except at *qSLT8.2* and *qSLT13.2*.

Ten QTLs were detected for SWT explaining 5.71 to 21.84% of PV (Table 2). One major and stable QTL *qSWT1.2*, was detected between markers Chr01_51855286 and Sat_414, in 2012 and 2017, and for the combined environments data. The *qSWT1.2* explained 11.25 and 15.77% of PV in RILs for years 2012 and 2017, respectively, and 21.84% of PV for combined environments data. In 2016, a major QTL *qSWT1.1* was detected near *qSWT1.2* at 159.5–166.5 cM, explaining 20% of PV. Stable QTL *qSWT4.3* was identified between markers Chr04_46382211 and Chr04_46508852, in 2012 and 2016, as well as for combined environments data with PV 5.71%–15.70%. Three QTLs, *qSWT4.1, qSWT9.1*, and *qSWT18.1*, were identified for combined environments data and the remainder were environment specific. Positive alleles for SWT were contributed by Fendou 16 at all QTLs detected for SWT, except *qSWT9.1* and *qSWT20.1.*

Composite interval mapping of seed shape traits identified 47 QTLs, 20 of which were shared with the QTLs identified by ICIM (Supplementary Tables 5, 6). All four stable QTLs, *qSLW2.1, qSLT2.1, qSWT1.2*, and *qSWT4.3*, were detected in CIM. This further confirmed the QTL mapping results.

### QTL Clusters for Seed Size and Shape Traits

**FIGURE 3:**
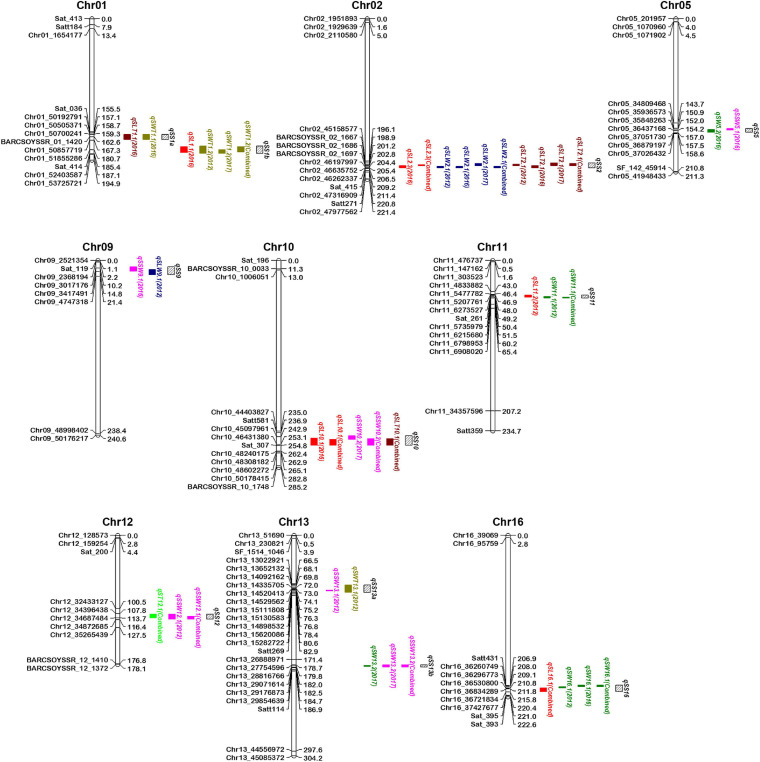
Map positions of quantitative trait loci (QTLs) for seed size and shape traits present in 11 QTL clusters on the genetic linkage map of the K099 × Fendou 16 RIL population. Distances in cM are indicated to the right of the linkage groups and names of markers are shown on the left. Markers of only in and around identified QTLs are shown on chromosomes. Positions of different QTLs are represented by colored bars on the right of chromosomes. QTL names are followed by the environment (year) in parentheses depicting year of QTL expression. QTL cluster positions are depicted by black bars with slanting lines on the right of chromosomes.

Most of the seed size and shape traits are positively or negatively correlated with each other perhaps because a QTL for one seed size and shape trait may be present in the same chromosomal region as QTL(s) of other trait(s). These QTLs for different seed size and shape traits may be either different or same locus due to random shifting and are referred to as integrated QTLs or QTL clusters. Of the total 80 QTLs identified for seven seed size and shape traits, 24 were located on 11 QTL clusters, distributed on nine chromosomes (Table 3 and Figure 3). These QTL clusters were designated as “*qSS*” (seed size and shape locus followed by chromosome number and their order), and were named *qSS1a, qSS1b, qSS2, qSS5, qSS9, qSS10, qSS11, qSS12, qSS13a, qSS13b*, and *qSS16*. Two QTL clusters, *qSS2* and *qSS10*, were composed of three QTLs each, while the other nine QTL clusters were composed of two QTLs each (Table 3). QTL cluster, *qSS2*, located between 198.5 cM and 205.5 cM on Chr.02 harbored two stable QTLs, *qSLW2.1*, and *qSLT2.1*, as well as a major SL QTL *qSL2.3*. QTL cluster *qSS10* located between 241.5 cM and 254.5 cM on Chr.10 harbored *qSL10.1*, *qSSW10.2*, and *qSLT10.1*, but none of them had shown consistent expression in the three growing environments.

**TABLE 3:** QTL clusters for seed size and shape traits in K099 × Fendou 16 RIL population.

**QTL cluster**	**QTL cluster**	**Individual QTLs**	**PVE^c^**	**Confidence**	**Environment^e^**
**name^a^**	**support interval (cM)**	**of cluster^b^**	**(%)**	**interval^d^ (cM)**	
*qSS1a*	159.5–166.5	*qSWT1.1*	20.06	159.5–166.5	2016
		*qSLT1.1*	13.45	159.5–166.5	2016
*qSS1b*	175.5–185.5	*qSL1.1*	11.7	176.5–184.5	2016
		*qSWT1.2*	11.25	175.5–185.5	2012
			15.77	180.5–185.5	2017
			21.84	176.5–183.5	Combined
*qSS2*	198.5–205.5	*qSL2.3*	16.65	202.5–205.5	2016
			19.85	201.5–202.5	Combined
		*qSLW2.1*	37	203.5–205.5	2012
			39.48	203.5–205.5	2016
			29.04	199.5–202.5	2017
			39.49	203.5–205.5	Combined
		*qSLT2.1*	13.7	200.5–202.5	2012
			20.68	202.5–205.5	2016
			21.41	198.5–202.5	2017
			23.21	199.5–202.5	Combined
*qSS5*	151.5–156.5	*qSW5.2*	9.48	152.5–156.5	2016
		*qSSW5.1*	17.63	151.5–153.5	2016
*qSS9*	8.5–19.5	*qSSW9.1*	9.72	8.5–14.5	2016
		*qSLW9.1*	6.91	12.5–19.5	2012
*qSS10*	241.5–254.5	*qSL10.1*	20.86	244.5–254.5	2016
			14.06	246.5–254.5	Combined
		*qSSW10.2*	17.79	241.5–246.5	2017
			10.98	245.5–254.5	Combined
		*qSLT10.1*	11.93	245.5–254.5	Combined
*qSS11*	47.5–51.5	*qSL11.2*	10.39	47.5–50.5	2012
		*qSW11.1*	9.34	49.5–51.5	2012
			5.79	50.5–51.5	Combined
*qSS12*	108.5–115.5	*qST12.1*	13.42	108.5–113.5	Combined
		*qSSW12.1*	11.13	108.5–115.5	2012
			20.38	111.5–115.5	Combined
*qSS13a*	68.5–78.5	*qSSW13.1*	11.14	75.5–76.5	2012
		*qSWT13.1*	14.77	68.5–78.5	2012
*qSS13b*	178.5–181.5	*qSW13.2*	35.21	179.5–180.5	2017
		*qSSW13.2*	16.2	178.5–181.5	2017
			12.86	178.5–181.5	Combined
*qSS16*	206.5–215.5	*qSL16.1*	4.85	210.5–215.5	Combined
		*qSW16.1*	14.74	208.5–210.5	2012
			21.47	206.5–208.5	2016
			23	206.5–208.5	Combined

*^a^Defined as neighboring QTLs within overlapped support (confidence) intervals. ^b^QTLs of various seed related traits with overlapping confidence interval. ^c^Phenotypic variance explained by QTL. ^d^The QTL confidence interval identified by 1-LOD drop from the peak LOD of QTL, in cM. ^e^Particular year in which QTL expressed.*

### Validation of Allelic Effect of Stable Locus *qSS2*

**FIGURE 4:**
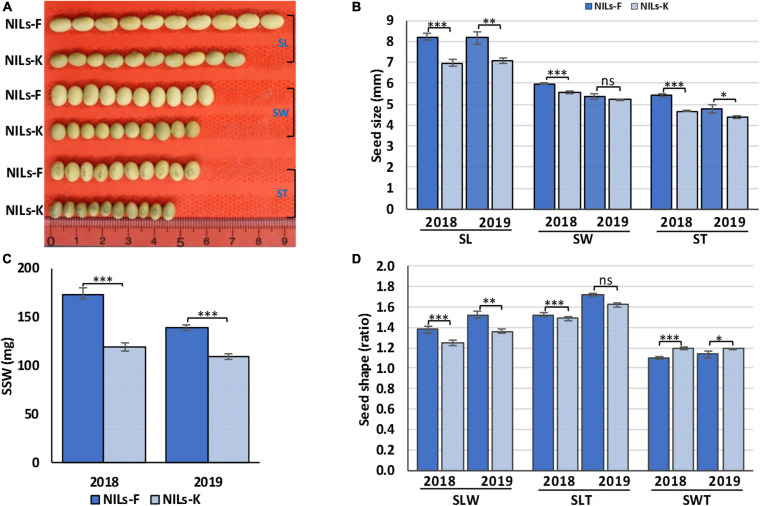
Seed size and shape phenotypes of near isogenic lines, NILs-F and NILs-K in 2018 and 2019, **(A)** phenotypic appearance, **(B)** SL, seed length; SW, seed width; ST, seed thickness, **(C)** single seed weight (SSW), and **(D)** seed shape traits (SLW, ratios of seed length-to-width; SLT, seed length-to-thickness; and SWT, seed width-to-thickness). Error bars represent means ± SD of three replicates. Asterisks indicate significant differences between NILs-F and NILs-K at 5% (^∗^), 1% (^∗∗^), and 0.1% (^∗∗∗^); ns indicates no significant difference at the 5% level in Student’s *t*-test.

The effect of QTL cluster *qSS2* on seed size and shape traits was validated using RHL-derived NILs, NILs-F and NILs-K. The NILs-F had the Fendou 16 homozygous genotype, and NILs-K had the K099 homozygous genotype at the *qSS2*. Genomic background difference analysis using 122 SSR markers identified 14 SSR markers as polymorphic, of which three were from the *qSS2* locus. Thus, other than the *qSS2* locus, the two NILs were differed at 9.01% of the background genome. Of the 11 QTL clusters, the NILs were differing at *qSS2* and *qSS10*. These two contrasting NILs were evaluated for all seven seed size and shape traits for 2 years, 2018 and 2019. NILs-F and NILs-K showed significant differences for SL and SLW in both years (*P* < 0.01) and for SLT in 2019 (*P* < 0.001) (Figure 4). They also showed significant differences in SSW, ST and SWT in both years (*P* < 0.01), and for SW in 2018 (*P* < 0.01; Supplementary Table 7). Although, *qSSW10.2* of QTL cluster *qSS10* was not stably detected in QTL analysis, the significant differences observed in SSW of NILs in both the years suggest that *qSSW10.2* was also stably expressed.

### Mining of Candidate Genes in *qSS2*

**FIGURE 5:**
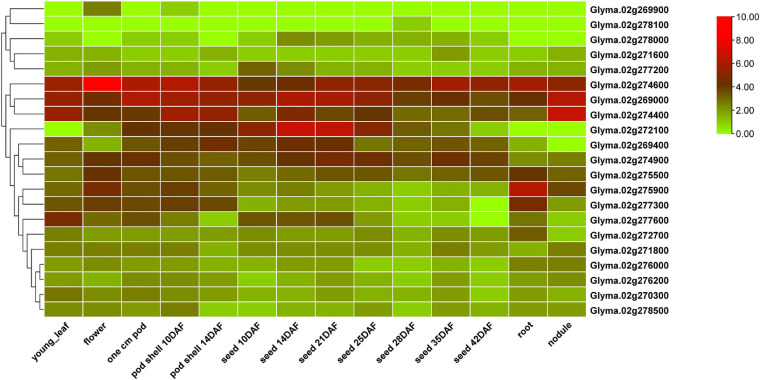
Heatmap visualization of relative gene expression in different tissues and developmental stages, for the candidate genes predicted in the *qSS2* physical interval. Normalized FPKM values are depicted on the log2 scale.

Quantitative trait locus cluster *qSS2* is flanked by BARCSOYSSR_02_1667 and Chr02_46197997. These markers are separated by a physical interval of ∼887 Kb. Based on the Glyma 2.0 assembly of Williams 82, 110 protein coding genes are predicted to be present in the genomic sequence of this physical interval (Supplementary Table 8). A total of 22 protein coding genes with gene function annotations for cell proliferation, differentiation, tissue morphogenesis and seed metabolism were selected as candidate genes (Supplementary Table 9). Twenty-one of the 22 predicted candidate genes are represented in the RNA-Seq atlas available in SoyBase. Expression analysis using RNA-Seq data for different soybean tissues showed that five of them have high expression specifically in pods and seed tissues at various developmental stages (Figure 5). Two *Glycosyl hydrolase* family members, *Glyma.02G269400* and *Glyma.02G272100*, were highly expressed in seed tissues and are associated with carbohydrate metabolism. *Glyma.02G274900*, a *translation initiation factor 2C* gene, was annotated with cytokinesis and several tissue differentiation terms. A cyclin-dependent protein serine/threonine kinase regulator activity gene, *Glyma.02G277600*, was highly expressed in seed tissues. *Glyma.02G277200*, encoding an *ATP synthase regulator*, was annotated with several organ morphogenesis terms, and was highly expressed in seed tissue 10 days after flowering. *Glyma.02G276000*, encoding *cyclin-dependent kinase C2*, was annotated for the vegetative to reproductive phase transition. Two *late embryogenesis abundant protein* genes (*Glyma.02G274400* and *Glyma.02G277300*), two *pentatricopeptide repeat* (*PPR*) *protein* genes (*Glyma.02G271600* and *Glyma.02G276200*) and a *protein phosphatase 2C* gene (*Glyma.02G278100*) are other potential candidates associated with seed development and morphogenesis, controlling various seed size and shape traits.

## Discussion

Seed size is one of the most important traits selected during domestication of soybean. In order to continuously improve seed yield and appearance in soybean, dissection of the genetic architecture of seed size and shape traits is imperative. Identification of genomic loci controlling seed size and shape traits and further characterization of genes underlying these loci can help in molecular designing of seed size and shape traits in soybean. Seed size and shape traits in soybean are highly complex and controlled by many loci as demonstrated in the earlier QTL mapping studies (Xu et al., 2011; Hu et al., 2013; Niu et al., 2013; Hina et al., 2020). Several QTLs for seed size and shape traits have been previously identified in soybean using bi-parental mapping (Hoeck et al., 2003; Salas et al., 2006; Xu et al., 2011; Hu et al., 2013; Che et al., 2014; Kato et al., 2014; Liang et al., 2016; Fang et al., 2017; Yang et al., 2017; Fujii et al., 2018; Cui et al., 2020; Hina et al., 2020; Li et al., 2020). Genome-wide association studies were also attempted to identify QTLs for seed size and shape traits in soybean (Hu et al., 2013; Niu et al., 2013; Fang et al., 2017; Li et al., 2019). Although, many QTLs were identified for seed size and shape traits in soybean, only a few were stable in different genetic backgrounds and environments (Hina et al., 2020). Further, looking at the vast diversity and complexity of these traits, additional characterization of these traits’ genetic underpinning in novel genetic resources is warranted. Fendou 16, the larger seed size parent used in our study, showed significant variation in SSW, SL, SLW, and SLT, by showing 21.5, 29.3, 30.7, and 36.7% higher values than small-seeded parent K099, whereas K099 showed 11.3% higher values of ST. Longer seed length with higher SLW and SLT are characteristics of wild species *Glycine soja*, while the opposite is the case for cultivated soybean. However, seed size and shape traits have evolved during domestication and it would be interesting to know whether the higher SLW and SLT ratio in Fendou 16 is related to wild soybean alleles or are newly acquired.

Genotyping by random amplicon sequencing-direct is a recently developed method for genotyping by sequencing using random primers for PCR amplification and next-generation sequencing, for identifying many highly reproducible genetic markers (Enoki and Takeuchi, 2018; Hosoya et al., 2019; Miki et al., 2020). Using GRAS-Di and SSR markers, a dense genetic map of K099 × Fendou 16 RIL population was developed. Using ICIM, we identified a large number of additive QTLs for seven seed size and shape traits. QTLs identified in two or more environments with PVE > 10% are considered as stable QTLs. In this study, we identified two stable QTL for seed size traits (*qSW8.1* and *qSW16.1*) and four stable QTLs for seed shape traits (*qSWT1.2, qSLW2.1, qSLT2.1*, and *qSWT4.3*), explaining a considerable phenotypic variation in these traits across multiple years. CIM further confirmed these stable QTLs including all QTLs of QTL cluster *qSS2*. Although, majority of seed size and shape trait QTLs were detected in CIM, the differences observed in the position of other QTLs may be due to small population size used in this study, as well as differences in the method of selecting background markers as cofactors and minimum map distance used to declare a new QTL in CIM compared to ICIM (Meng et al., 2015). The large effect and stable QTL *qSLW2.1* was earlier reported for the association with SLW (Niu et al., 2013), and ST and SLT (Fang et al., 2017), in association mapping studies. In the present study, we found that *qSLW2.1* also control SL and SLT, as the major QTLs *qSL2.3* and *qSLT2.1*, were co-localized in the same genomic region of *qSLW2.1*. Thus a major gene in this genomic region affects SL, SLW and SLT, which is also evident from their high positive correlation. This major locus, a QTL cluster for seed size and shape traits in soybean, has been named as *qSS2*. The NILs contrasting for *qSS2* showed significant differences for various seed size and shape traits evaluated for 2 years’ growing environments, confirming *qSS2* as a major and stable locus. In the present study, no stable QTL was identified for SSW at *qSS2* but a SSW QTL *qSSW2.1* was identified near *qSS2*. The two NILs also differed at *qSSW2.1* and *qSSW10.2*, perhaps explaining significant difference for SSW. The interaction of both these QTLs with *qSS2*, might be contributing to significantly large effects on seed size and shape traits, therefore further studies are required to dissect the effect of individual and multiple QTL clusters on seed size and shape traits.

Clustering of QTLs for seed size and shape traits has been frequently reported elsewhere. Xu et al. (2011) reported co-localization of several seed size and shape QTLs on Chr.06 and Chr.10, in the direct and reciprocal cross of Lishuizhongzihuang × Nannong 493-1. QTL cluster identified on Chr.06 was fine mapped and eight candidate genes were predicted (Xie et al., 2014). Hina et al. (2020) reported co-localization of several seed size and shape QTLs in four QTL hotspots on Chr.06, Chr.10, Chr.13, and Chr.20, in two RIL populations developed from the crossing of higher seed size genotype Nannong1138-2 (N) with cultivated soybean varieties Zhengxiaodou (Z) and Kefeng35 (K3). Recently, Cui et al. (2020) identified 30 significant QTLs for six seed size and shape traits in a RIL population of BX10 × BD2. Among these QTLs, co-localization of seven QTLs for five traits was found at one locus, named *qSS3*. The regular reports on co-localization of QTLs for seed size and shape traits are probably due to high correlation among various seed size and shape traits.

An SW QTL, *qSW19.1*, was identified in the nearby genomic region earlier reported by Xu et al. (2011) and Niu et al. (2013). Two stable QTLs for SWT, *qSWT1.2* and *qSWT4.3*, were not reported earlier and are newly identified. Using high-density mapping in two RIL populations, Hina et al. (2020) identified three stable QTLs for SL on Chr.04, Chr.09, and Chr.13, one for SW on Chr.13 and two for ST on Chr.06 and Chr.13. Among the seed shape traits, Hina et al. (2020) identified three stable QTLs for SLW on Chr.06, Chr.19, and Chr.20, two for SLT on Chr.10 and Chr.20, and two for SWT on Chr.02 and Chr.08, but none of them was present in the overlapping genomic regions of stable QTLs identified in the present study. Notably, only three stable QTLs (*qSL-9-1_*ZY,K*__3__*N*_, qSLW-19-1_*K*__3__*N,ZY*_*, and *qSWT-2-1_*K*__3__*N,ZY*_*) were common between two RILs evaluated by Hina et al. (2020). Cui et al. (2020) also identified one stable QTL named *qSS3*, on Chr.03 explaining 7.3 to 26.2% of PV for five seed size and shape traits, in a RIL population tested in three growing locations. The *qSS3* was validated in RHL-derived NILs (Cui et al., 2020). These stable QTLs with significantly large effect phenotypic variance contribution are suitable for practical application in soybean breeding to improve seed size and appearance traits. The Fendou 16 will serve as a vital genetic resource for the introgression of favorable alleles of SL, SLW, and SLT traits in soybean.

The identified stable QTLs will pave the way for positional cloning of genes regulating seed size and shape traits in soybean. Several QTLs and genes controlling seed size traits have been cloned in other crop species, but similar studies are lagging in soybean (Mao et al., 2010; Li et al., 2011; Ma et al., 2015; Xu et al., 2015; Lu et al., 2017; Nguyen et al., 2020). The physical interval of the *qSS2*, spans ∼887 Kb and harbors 110 protein coding genes. Among them, 22 genes were annotated with cell differentiation, tissue morphogenesis, signal transduction, carbohydrate metabolic process and fatty acid biosynthetic process, and might be involved in regulating various seed size and shape traits. Candidate genes were further prioritized based on the RNA-Seq gene expression data of seven seed tissues. *Glycosyl hydrolase family* members are involved in lignin and cellulose degradation pathway in microbes, but not characterized in plants (Davison and Blaxter, 2005). Three *glycosyl hydrolase family* genes were present in *qSS2*, of which two had shown high expression in seed tissues. *Glyma.02G275500* encodes a 5′-AMP-activated protein kinase beta subunit. The gene encoding *SNF1-related protein kinase-1* is plant homolog of mammalian *5*′*-AMP-activated protein kinase beta-2 subunit protein*, which controls cell proliferation in *Arabidopsis* (Guérinier et al., 2013). *Glyma.02G276000* encodes a cyclin-dependent kinase C2. Cyclin-dependent kinases play an important role in tissue differentiation. Loss of a c*yclin-dependent kinase C* showed an altered size of leaves and stomatal patterning in *Arabidopsis* (Zhao et al., 2017). Two *PPR protein* genes (*Glyma.02G271600* and *Glyma.02G276200*) were present in *qSS2*. Genes encoding PPR have been shown to affect kernel size in maize (Huang et al., 2020) and seed development in maize and rice (Li et al., 2014). A *protein phosphatase 2C* gene in soybean controls seed weight in soybean (Lu et al., 2017). *Glyma.02G278100*, encoding a *protein phosphatase 2C*, is a potential candidate gene for seed size and shape at *qSS2*. Cui et al. (2020) delimited a QTL hotspot, *qSS3*, to a ∼1,126 kb region, and 123 genes were predicted in the candidate genomic region. Hina et al. (2020) predicted 88 candidate genes for seed size and shape traits in 4 QTL hotspots clustered on Chr.06, Chr.10, Chr.13, and Chr.20. These candidate genes are stepping stones for identifying causal genes for seed size and shape traits in soybean.

## Conclusion

We have identified six stable QTLs for seed size and shape traits accounting for significant phenotypic contributions across multiple years, besides identifying several other environment-specific QTLs. QTL clusters identified for various seed size and shape traits indicate many strong inter-relationships among these traits. Major genomic locus *qSS2*, harboring three major QTLs was validated in NILs. Information on predicted candidate genes for *qSS2* is useful for identifying causal genes determining seed size and shape traits in soybean. This study will assist in breeding programs aiming to improve seed size and shape traits in soybean.

## Data Availability Statement

All datasets presented in this study are available in the article and associated Supplementary Material.

## Author Contributions

DX designed the experiments. GK and DX carried out the experiments and analysis, and wrote the manuscript. Both authors contributed to the article and approved the submitted version.

## Conflict of Interest

The authors declare that the research was conducted in the absence of any commercial or financial relationships that could be construed as a potential conflict of interest.

## Footnotes

1 https://www.legumebase.brc.miyazaki-u.ac.jp/

2 https://www.ars-grin.gov/npgs/

3 https://www.soybase.org/

## Supplementary Material

Supplementary Table 1 Phenotypic differences between two parents of recombinant inbred lines (RIL) population, K099 and Fendou 16.

Supplementary Table 2 Descriptive statistics of seed size and shape traits evaluated in recombinant inbred line (*n* = 94) population developed from cross K099 × Fendou 16.

Supplementary Table 3 Linkage map information of 1662 markers mapped in K099 × Fendou 16 RILs.

Supplementary Table 4 Statistical details for linkage map of K099 × Fendou 16 RILs.

Supplementary Table 5 Seed size and shape related QTLs identified in RILs by composite interval mapping (CIM).

Supplementary Table 6 Comparisons of QTLs for seed size and shape related traits identified by inclusive composite interval mapping (ICIM) and composite interval mapping (CIM).

Supplementary Table 7 Phenotypic differences for seed size and shape traits between two NILs for *qSS2*, NILs-K and NILs-F.

Supplementary Table 8 Predicted protein coding genes in the physical interval of q*SS2* and their annotation information according to Williams 82 assembly Gmax version 2.0 in SoyBase.

Supplementary Table 9 Predictive candidate genes in *qSS2*.
